# Weight change among repeat participants of an Aboriginal community-based weight loss program

**DOI:** 10.1186/s12889-020-09086-6

**Published:** 2020-06-26

**Authors:** Erika Bohn-Goldbaum, Aaron Cashmore, Rose Fonua, Andrew Milat, Kate Reid, Leah Shepherd, Adrian Bauman, Anne C. Grunseit

**Affiliations:** 1 The Australian Prevention Partnership Centre, Sydney, New South Wales Australia; 2 The University of Sydney, Sydney School of Public Health, Camperdown, New South Wales Australia; 3 Centre for Epidemiology and Evidence, NSW Ministry of Health, St Leonards, New South Wales Australia; 4 School of Public Health and Community Medicine, University of NSW, Kensington, New South Wales Australia; 5 NSW Office of Preventive Health, St Leonards, New South Wales Australia

**Keywords:** Weight loss, Repeat participation, Aboriginal Australian

## Abstract

**Background:**

Community-based weight loss programs may have potential to address overweight and obesity at the population level. However, participation patterns and individual outcomes from these programs are understudied. This study examined repeat participation patterns and participant weight change between contests over seven years of an Aboriginal Australian team-based program in order to identify (1) predictors of repeat participation and (2) associations with weight change between contests.

**Methods:**

Data for the 12 contests from 2012 to 2018 were merged, with probabilistic record matching. A total of 7510 enrolments were registered for the 12 contests, representing 4438 unique people. Contest lengths varied from 10 to 16 weeks in duration. Non-repeat participants were those who only competed once in the program by the end of 2018, and repeaters were those who competed in at least two contests. Associations between repeat participation and participant baseline (i.e., first participation occasion) characteristics, change in diet and physical activity and percent change in weight during the first participation occasion were examined using crossed random effects (for person and team) regression adjusted for exposure to the program. Weight percentage change between contests was calculated for consecutive participation occasions occurring at least three months apart, converted to percent change per month. Weight change was regressed on number of repeat participation occasions adjusted for age, gender, baseline weight at first participation occasion, and weight percent change in the immediately preceding contest.

**Results:**

One-third of the 4433 participants participated more than once, with women more likely than men to repeat. A 1% reduction in weight during a competition was associated with an increase in weight of 0.05% per month between competition end and subsequent participation. Regain was smaller the heavier participants were at their first participation.

**Conclusions:**

While individuals benefit from weight loss through program participation, strengthening strategies for weight loss maintenance within or following the program could improve long-term weight outcomes and reduce weight cycling.

## Introduction

Weight loss interventions among Aboriginal Australians have proven effective in reducing weight and non-communicable disease risk [1–5]. Effective interventions are particularly needed for a population of whom over two-thirds are overweight or obese and whose mortality rate is 1.6 times that of non-Indigenous Australians [6]. Community-based programs offer easy and affordable access and have potential to both strengthen community ties and improve physical health [7–9]. Outcomes from community-based weight loss programs can be difficult to establish due to the variety of program designs and reporting inconsistencies [10]; never-the-less, weight reduction and reduced risk of non-communicable disease have been demonstrated in other populations [11–14]. For Aboriginal Australians there is limited but positive evidence of effectiveness for community-based programs [15].

Losing excess weight has health benefits for cardiovascular and other risk factors [16] but the health effects from any weight regain are less clear, with some studies suggesting loss of health benefits attained through weight loss [17], increased health risks [18], or improved risks relative to those who remain at a stable weight [19] or maintain the lost weight [20]. Whilst there is a large literature documenting weight loss, there are far fewer studies examining maintenance of weight loss, with most finding poor maintenance rates and regain common [21, 22]. Additionally, little is known about weight maintenance among Aboriginal Australians.

Attendance in an intervention and in any post-intervention program maintenance phase can aid weight loss and maintenance [23, 24]. Repeat program participation may also improve weight outcomes [11, 25, 26], particularly if the repetition is consecutive [27]. However, repeat participation may also indicate repeated loss/regain (weight cycling) if weight loss is not maintained in the interim, and weight cycling may be detrimental to health [28–32]. Thus, investigating characteristics of repeat participants and their weight changes in and between programs has potential public health implications.

Team-based weight-loss competitions are often used in wellness programming as they have wide reach for population impact [33]. There are some successful, repeated, team-based, community weight-loss competitions, but individual outcomes of community competitions are infrequently evaluated in peer-reviewed literature [33]. There are three notable exceptions based in the United States of America. Repeat participants in Shape Up Rhode Island, an annual 16-week state-wide team-based competition, were more likely to be older (> 40 yrs), obese and meet guidelines for attaining moderate physical activity (PA) and vegetable consumption compared to non-repeaters [27]. Completion rates and weight loss correlated with social influence [12, 14], and both more minutes/week in PA and more steps/day correlated to weight loss [14]. In the second study, employees in an annual 5-week team-based competition at a Michigan hospital lost weight, with their outcomes differing with participation patterns over the program’s 5 years; over two-thirds of the employees gained weight between contests [34]. In the third study, individuals’ weight changes within and between an annual 14-week community competition in Texas were documented [11]. Average weight loss among first-time participants who lost weight during their first participation was approximately 5% and lessened with each subsequent participation; weight regain between first and second participations was approximately 3%, with non-consecutive repeat participants experiencing more regain [11]. Factors associated with weight changes included gender, starting weight, and the number of repeat participations [11].

We recently evaluated a community-based team weight loss competition, the NSW Aboriginal Knockout Health Challenge (KHC) [15], a program open to Aboriginal Australians and Torres Strait Islander peoples aged 16 years or older. KHC contests have been held once each in 2012 and 2013, and twice per year since 2014. Contest lengths have varied from 10 to 16 weeks in duration. The time between the start of the first (or only) contest in 1 year and the end of the previous contest ranged between 178 and 245 days, with shorter periods (17–94 days) between contests held in the same year. (Contests lengths and dates can be found in the additional file, supplemental Table 1). The program has been described in detail elsewhere [35]; briefly, teams of 20 or more persons compete to lose weight through PA and healthy eating. Participants may enrol as a member of a team at the beginning of any contest and teams self-determine their activities to support healthy lifestyle behaviours. The program capitalizes on Aboriginal Australians’ values (e.g., community) and is structured for prize money to be directed to environmental and socio-economic factors which disproportionally affect Aboriginal Australians and contribute to their poor health [6]. Our recent evaluation of the KHC found increased participation and significant average weight loss [15] but did not explore within-individual outcomes. Therefore, we analysed participation patterns, particularly repeat participation patterns, and participant weight change between contests over 7 years (2012–2018) of the KHC. The study aims were to identify (1) predictors of repeat attendance and (2) the effects of repeat attendance on weight change. Ethics approval for the secondary analysis was provided by the Aboriginal Health and Medical Research Council (Project 1125/15) and the University of Sydney (2019/425).

## Methods

### Data collection and treatment

Participant outcomes are measured pre-post contest for prize allocation; written consent (provided by the participant, and, for those under 18 years of age, by their parent/legal guardian with the underage participant providing written assent) allows use of the data for prize calculation and research purposes. Participants join teams by submitting data and consent to a team manager; data are collated and sent to a central database. Data comprise name, date of birth and gender as well as objective measures of weight (to nearest 0.1 kg) and height (cm) which were collected by a health professional. From 2013, self-reported current smoking status, fruit and vegetable intake (servings of each on a typical day), and from 2014, PA (frequency in last 7 days of 20 min or more vigorous PA, 30 min or more of walking, and 30 min or more of moderate PA) were also recorded using validated questions [36]. At the conclusion of the contest, participants’ weights are recorded by a health professional and self-reported lifestyle risk factors are reported via questionnaire using the same questions as at registration.

Data for the 12 contests were merged, with probabilistic record matching by participant name, sex and date of birth through an independent data linkage agency (The Centre for Health Record Linkage – http://www.cherel.org.au/; 0.05% false positive rate). The start and end dates of the 12 contests were then merged with the contest data.

### Participants

A total of 7510 enrolments were registered for the 12 contests from 2012 to 2018, representing 4438 unique people. Six records (*n* = 5 people) were excluded because the participant was aged younger than 18 years and did not have parental consent for their data to be used, leaving 4433 unique people with 7504 enrolments.

### Primary outcomes

This study had two primary outcomes: to identify (1) predictors of repeat participation and (2) associations with weight change between contests. Non-repeat participants were those who only competed once in the KHC by the end of 2018, and repeaters were those who competed in at least two contests. In order to account for the changing probability of repeating across time (because those whose first participation is later in the KHC series have less opportunity to repeat than those who start earlier [37]) an exposure variable was calculated as the number of contests between first and second occasion of participation or, for non-repeaters, the number of contests from first participation through to the 12th contest. Weight percentage change between contests was calculated for consecutive participation occasions which occurred a minimum of 3 months apart (i.e., between one contest’s end and start of the next contest the individual competed in). The end weight of the previous contest was subtracted from the start weight of the next and the difference divided by the end weight of the previous. For example, a participant weighing 100 kg at the start of contest 7 and 95 kg at the end of contest 6 (~ 7 months prior), would have a change of 5.3%; a positive number therefore demonstrates a weight gain between contests. This was converted to a rate per month by dividing by the number of months between the two contests using the contest start and end dates.

### Data analysis

Participation patterns were ranked from most to least common among the 4433 unique participants.

Further exclusions of the 4433 participants occurred at analysis. Two records were excluded from analyses as their weight change exceeded limits (e.g., a weight reduction greater than 30%) used previously in a weight management program of a similar duration as the KHC [23]. All other weights were within limits (leaving 4432 people with 7502 records). For likelihood of repeat participation, data comprised individuals who had formed teams of ≥20 persons at their first participation occasion (leaving 4312 people) and those whose first participation was prior to contest 12 (*n* = 4104 people). For analysis on weight changes between competitions, analysis was limited to participants who had 2 or more participation occasions (leaving 1532 people with 3071 records) and to those occasions where contest participation had a minimum three-month gap (leaving 1132 people with 1794 records). Further, a complete case approach was used for all analyses (i.e., relevant records with missing data were excluded).

The associations between repeat participation and participant baseline (defined as first participation occasion) characteristics, change in diet and PA, and percent change in weight during the first participation occasion were assessed using bivariate analyses. Baseline characteristics included gender (male, female), age (years), weight (kg), fruit and vegetable intake (per serve), and minutes of vigorous and moderate PA and walking. Fruit and vegetable intake were also categorised dichotomously as meeting (or not meeting) current dietary recommended levels of two serves of fruit and five serves of vegetables [38]. PA was categorised dichotomously as having (or not having) adequate PA (defined as three or more vigorous sessions/week; or five or more walking or moderate sessions/week; or 1–2 vigorous sessions/week and 3–4 walking or moderate sessions/week according to previous procedures) [39]. Associations between repeat participation and change in diet and PA (calculated by subtracting baseline from post-intervention scores for each contest) and percent change in weight during the first participation occasion were further adjusted for gender and age, as these variables have been shown previously to affect contest outcomes [15]. Crossed random effects Poisson models with random effects for person and team were used to account for the clustering of observations and were adjusted for the exposure to the KHC. Crossed random effects were used for all analyses to account for the clustering of observations within person and within team because participants did not uniquely nest within teams but changed teams across contests [40]. For repeat participation analyses only, results are reported as rate ratios (RR) for at least one occasion of repeat participation.

The analysis of the effect of repeat participation on weight percent change between contests was restricted to sequential participation occasions at least 3 months apart (to allow for weight regain following weight loss [22]). Cumulative repeat participation was operationalised as a categorical variable ranging from 2 to 6+ participation occasions to examine non-linear effects; 6–12 cumulative participation occasions were collapsed to form the category 6+ due to small numbers. Covariates were age, gender, baseline weight at first participation occasion, and weight percent change in the immediately preceding contest. Linear crossed random effects models were used with random effects for person and team. Results are presented as percent change in weight per month for a one unit increase in the independent variable.

All analyses were conducted using Stata 15.1 (College Station, TX, USA).

## Results

### Participation patterns

Over the 12 contests comprising the challenge, 4433 individuals participated, yielding a total of 7504 participation occasions. Participant baseline characteristics and participation patterns are shown in Tables 1 and 2, respectively. (An additional file contains further contest and participation characteristics not shown here.) Single-contest participation was the most common pattern, with 2901 (65.4%) participants. Contest 5 had the highest single-participation participants (*n* = 384, 8.7%) and contest 8 had the lowest (*n* = 75, 1.7%). Among those who had participated more than once (*n* = 1532, 34.3%), participating twice (*n* = 816, 18.4%) was the most common frequency and no individual competed in all 12 contests. The number of days between the official end of one contest to the start of the next contest ranged between 17 and 245 days.

**Table 1:** Participant baseline characteristics

Baseline characteristic	All participants n (%) or mean ± sd	Repeaters only n (%) or mean ± sd
Total	4433 (100%)	1532 (100%)
Gender (male), n = 4417	1174 (26%)	352 (23%)
Age (year), n = 4387	38.3 ± 12.95	38.2 ± 12.15
Weight (kg), n = 4403	96.6 ± 23.52	97.1 ± 22.62
Number of fruit serves/day, n = 3819	1.4 ± 1.17	1.45 ± 1.16
Number of vegetable serves/day, n = 3932	2.1 ± 1.52	2.1 ± 1.48
Number of sessions/week vigorous PA, n = 3515	1.8 ± 2.40	1.9 ± 2.68
Number of sessions walking/week, n = 3524	2.3 ± 2.71	2.2 ± 2.41
Number of sessions/week moderate PA, n = 3494	1.6 ± 2.19	1.6 ± 2.04

Baseline was defined as an individual’s first participation occasion. Analysis included data for 4433 participants, of whom 1532 participated more than once, using a complete case approach.

**Table 2:** Individuals’ participation patterns

# times competed	Type of repetition	n	Relative %	% total
1	n/a	2901	100	65.4
2	All	816	100	18.4
	Consecutive participation	489	59.9	
3	All	349	100	7.9
	3 consecutive participation	99	28.4	
	≥2 consecutive participation	295	84.5	
4	All	159	100	3.5
	4 consecutive participation	40	25.2	
	≥2 consecutive participation	156	98.1	
5	All	88	100	1.9
6	All	55	100	1.2
7	All	28	100	.6
8	All	22	100	.5
9	All	7	100	.2
10	All	5	100	.1
11	All	3	100	.1
Total		4433	100	99.8

Within low frequency repeaters, consecutive participation is further described with percentages relative to the sample repeating. Participation percentage does not equal 100% due to rounding (n = 4433).

The participation pattern for most repeaters included some consecutive contest participation (n = 1147, 74.9% of repeaters). The most frequent pattern was competing in contest 11 and contest 12 (n = 85, 10.4% of two-time participants). Consecutive participation was more frequent (n = 489, 59.9% of two-time participants) than skipping one or more contests in between. The number of two-time participants decreased with increasing gap between contests, with just 4 persons (0.5% of two-time participants) competing in only the first and last contests. Among those competing three or four times, most (84.5 and 98.1%, respectively) had at least two consecutive participations and few (25.2 and 28.4%, respectively) had no skipped contest between their first and last participation.

### Likelihood of repeating

Table 3 shows the bivariate rate ratios (with 95% CI) of repeating at least once for characteristics of participants on their first occasion of participating, adjusting for the number of possible contests they could enter as described in the methods. Among the demographic, weight and behaviour measures only gender and, marginally, amount of vigorous PA showed significant relationships with the likelihood of repeat participation. Female participants had a 34% higher likelihood of repeating, and the likelihood of repeating increased by 2% for every extra session of vigorous PA reported at registration in the first participation. Neither the number of fruit nor vegetable serves nor PA behaviour bore much relation to repeat participation. Further, meeting dietary and PA recommendations at first participation start, versus not meeting these, was not related to repeat participation (see additional file, supplemental Table 4).

**Table 3:** Rate ratio for repeat participation in the KHC for baseline characteristics (bivariate analyses)

Baseline characteristic^*a*^ (reference unit)	Rate ratio (RR)^*b*^	95%CI	p-value
Gender (male)	1.34	(1.18, 1.52)	< 0.001
Age (10 years)	0.99	(0.95, 1.03)	0.588
Weight (5 kg)	1.00	(0.99, 1.01)	0.538
Number of fruit serves/day (1 serve/day)	1.01	(0.96, 1.06)	0.720
Number of vegetable serves/day (1 serve/day)	0.99	(0.96, 1.03)	0.793
Number of sessions/week vigorous PA (1 session/week)	1.02	(1.00, 1.05)	0.052
Number of sessions/week walking (1 session/week)	0.99	(0.96, 1.01)	0.229
Number of sessions/week moderate PA (1 session/week)	1.01	(0.98, 1.04)	0.528

^*a*^Baseline defined as participants’ first participation occasion. ^*b*^per 1 reference unit increase or compared to categorical reference unit. As a complete case approach was used, the n for each analysis varies: gender = 4089, age = 4063, weight = 4076, fruit serves = 3494, vegetable serves = 3607, sessions vigorous PA = 3196, sessions walking = 3202, and sessions moderate PA = 3175

Table 4 shows the rate ratio for repeating given participants’ outcomes in their first contest, adjusted for age and gender and possible repetition occasions. The likelihood of repeat participation increased with increased weight loss and with a higher number of sessions of walking and moderate PA as measured at the end of their first occasion of participation. Specifically, the likelihood of repeating increased by 3% per percentage of registration weight lost and by 7 and 6%, respectively, for each session increase of walking and moderate PA. There were marginally significant results for number of vegetable serves (p = 0.071) and of vigorous PA sessions (p = 0.065), both demonstrating an incremental effect as the number increased.

**Table 4:** Rate ratio for repeat participation in the KHC for outcomes of participants’ first participation occasion

Change in first participation occasion	Rate ratio (RR)^a^	95%CI	p-value
Weight % lost (1%)	1.03	(1.02, 1.05)	< 0.001
Number of fruit serves/day (1 serve/day)	1.03	(0.98, 1.09)	0.237
Number of vegetable serves/day (1 serve/day)	1.05	(1.00, 1.11)	0.071
Number of sessions/week vigorous PA (1 session/week)	1.04	(1.00, 1.08)	0.065
Number of sessions/week walking (1 session/week)	1.07	(1.03, 1.11)	< 0.001
Number of session/week moderate PA (1 session/week)	1.06	(1.02, 1.10)	0.005

^a^ per 1 reference unit increase, and further adjusted for sex and age and accounting for possible repetition occasions. As a complete case approach was used, the n for each analysis varies: weight = 2397, fruit serves = 1840, vegetable serves = 1201, sessions vigorous PA = 992, sessions walking = 992, sessions moderate PA = 974

### Impact of repeat participation on weight between contests

**Fig. 1:**
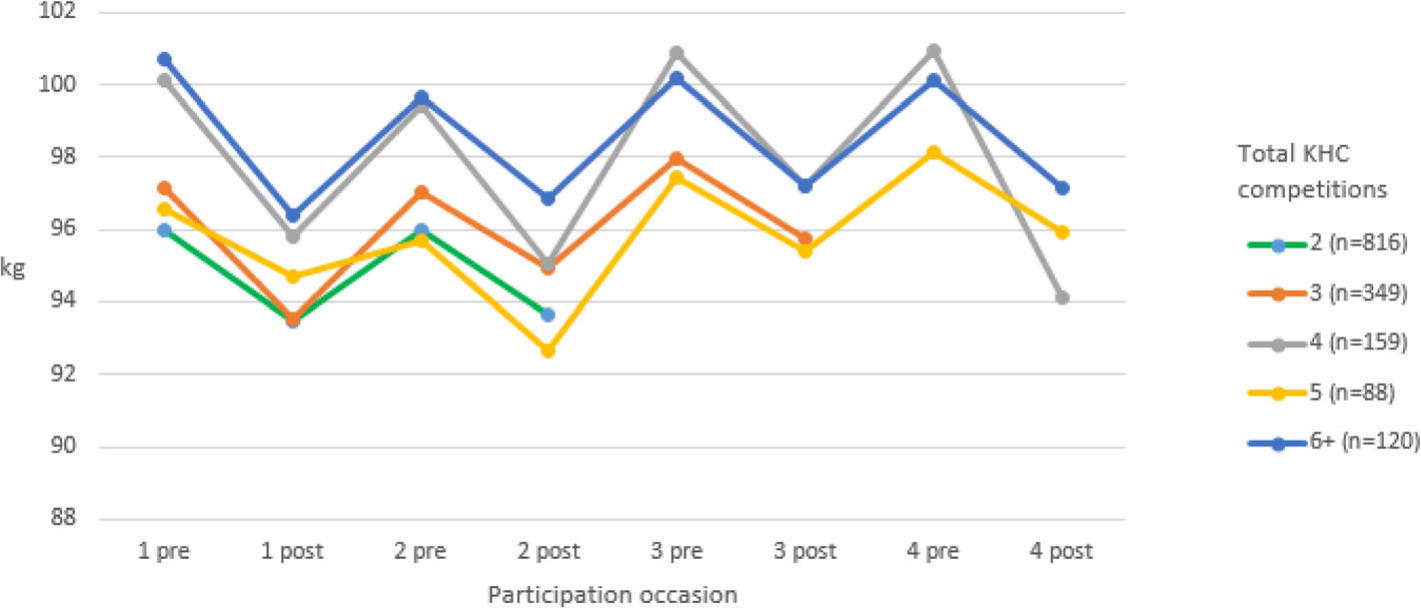
Weight changes over four participation occasions. Participants’ raw mean weight at start and end of each contest occasion, for those competing two or more times, censored at 4 participations; *n* = 1532

Figure 1 shows participants’ raw mean weight at start and end of each contest occasion, for those competing two or more times. It appears that there is a decrease in weight during each contest and a subsequent increase during the time before the subsequent participation.

Table 5 shows weight changes between contests, adjusted for age, gender, percentage weight lost during the previous contest, the starting weight on the first occasion of participating, and the number of participation occasions at that point. Due to small numbers, participation occasions 6–11 were combined. Holding all else equal, for every percentage increase in the amount of weight lost, there was a 0.05 percentage gain per month in between contests. However, for every kilogram heavier the participant was on their first occasion of competing, there was a small (0.002) decrement in the percentage weight regain. There appeared to be somewhat of a dose-response effect for repeat participation whereby the amount of weight gain between contests increased as the number of participation occasions increased. For example, predicted percentage gain per month generated using average values on all other covariates showed that the weight gain per month after two participation occasions was 0.30%, after four was 0.57%, and after six was 0.70% per month (data not shown). A supplementary analysis using participation as a continuous variable found that the average increase in weight percent per month was 0.10 (.06, .13, p < .001) per participation occasion.

**Table 5:** Adjusted percentage weight change per month between contests at least three months apart (*n* = 1107)^1^

Variable (reference category)	Average change in between-contest weight change (%)	95%CI	p-value
Age in years	−0.001	−0.005, 0.002	0.475
Gender (male)	0.03	−0.80, 0.13	0.636
Weight, % change during last competition	0.05	0.04, 0.06	< 0.001
Weight, in kg at first repeat occasion	−0.002	−0.004, − 0.0002	0.025
Number of participation occasions (two)			< 0.001^2^
3 occasions	0.15	0.05, 0.25	
4 occasions	0.27	0.11, 0.44	
5 occasions	0.38	0.20, 0.57	
6–11 occasions	0.39	0.20, 0.59	

^***1***^ Missing values were excluded using a complete case approach. Analysis included data for 801 people across 1107 records. ^2^p-value is for joint effect

## Discussion

This study analysed participation patterns and participant weight change between contests over 7 years (2012–2018) of a community-based weight loss program for Aboriginal Australians. About one-third of participants repeated the community weight loss program. Gender was a strong predictor of whether someone participated more than once, with behavioural measures contributing minimally to this prediction. Between competitions, repeat participants regained some of the weight they had previously lost, with smaller regain the heavier a participant was when they first joined the KHC.

### Participation

The KHC is a long-running program with increasing appeal [15]. While the most common participation pattern in the KHC was a single participation occasion, repeaters accounted for about one-third of the participants. In our study, almost no baseline characteristic predicted repeat participation. The observed increased likelihood of repeat participation among women contrasts with results from studies of online PA and other behaviour change programs which found no gender difference [27, 41] or a higher likelihood for repeat participation among men [41]. In those studies and this, males comprised a minority of the study population, consistent with findings that men are less likely to seek assistance for weight loss [42]. Reports of age [27, 41] predicting repeat participation contrast with our findings but could be explained by the internet-based program format of those programs. Previous research found attaining sufficient vigorous PA did not determine likelihood of repeat participation [27],whereas here the amount of vigorous PA reported at baseline was marginally (*p* = .052) related to the likelihood of repeat participation. Again, the different program formats may explain variabilities in health behaviours as participation predictors. Additionally, the KHC serves a unique population (Aboriginal Australians) and its incorporation of their values (e.g., reflecting the strong value in family and community by having participation based on teams) may influence repeat participation likelihood [43]. Investigating participant motives for involvement in the KHC and satisfaction with the program format may elucidate why a gender difference in repeat participation exists and help tailor the program to maximize its reach.

Whilst we identified predictors of repeat participation, we did not measure goals and expectations. We did model the likelihood of repeating on changes in lifestyle outcomes during participants’ first participation occasion. Greater increases in walking and moderate PA sessions and greater weight loss were associated with increased likelihood of repeating by between 3 and 7%. Previous research has also found higher levels of participation [11, 25, 26] are associated with improved weight outcomes. Identifying whether actualized behaviour changes were participant goals could provide insight into the relationships between goals, behaviour changes and repeat participation. Further, there are a variety of other motives for program participation, including health concerns and social support [44], and these directly relate to the secondary goals of the KHC. Motives may differ between first-time and repeat participants in physical activity events [45]. Investigating repeat and non-repeat participant motives may be helpful for further program development or to elucidate other strategies needed to combat weight loss.

### Weight change between repeat contests

Repeat participants gained weight between contests at a rate of 0.05% body weight per month. Similarly, weight regain, of approximately 3%, was also found during the 40-week length gap between first and second participations in another community-based weight loss competition [11]. That weight is regained between contests, increases with each participation occasion, and correlated with a higher percent weight lost during the program suggests weight cycling may be a problem among repeat participants. Figure 1 also lends itself to such an interpretation. Positively, regain may be less of a problem for heavier participants (here and in the American program [11]), which suggests community-based programs may have a positive health impact among obese individuals. Further, this type of community program may confer additional benefit for weight loss through its team-based format as weight loss can be clustered within teams [12] and team-based participation may increase the amount weight lost [11, 14]. On the other hand, team-based participation may also increase the likelihood for regain as the social support available from teammates during participation disperses [11]. Social support has been implicated as having a potential role in weight loss [14] while individual coping skills, such as self-monitoring [46, 47] and emotional control [48], may be key to maintenance. Thus, structuring a community program to maximize the benefits of social support during competition while teaching individual skills for maintenance may be important to minimize weight cycling. Investigating coping skills of repeaters and non-repeaters may indicate where skill differences exist and may be more valuable for weight maintenance among this population.

Males comprise an equal proportion of the overweight/obese Aboriginal population [49]. They may be less likely to lose weight in the KHC [35] and have lower participation and probability of repeating than females, but our results indicate they are no more likely than females to regain lost weight. Similarly, the observed gender difference in weight regain disappeared with participation cycles in the American program [11] and a recent systematic review found participant demographics were not associated with weight loss maintenance [22]. Therefore, addressing avenues to increase male participation and weight loss success in the KHC could have long-term population health benefits.

That weight increases with time between participation occasions reinforces the need for weight maintenance support. Evidence suggests self-monitoring assists with weight maintenance [46, 47], so incorporating this as part of the program may mitigate weight regain among participants. Notably, the 2013 KHC contest included a maintenance phase comprising individual (e.g., monthly weigh-ins) and team-based (e.g., team sports carnival competition) components [35]. While few KHC participants were evaluated in the maintenance phase, the results were promising: the mean weight at contest end and 9 months thereafter were comparable [35]. There is some evidence that web-based programs can assist maintenance as well, particularly in the short-term [50], and the KHC already has a social media presence which could act as a platform for post and/or between competition weight loss maintenance support. Weight loss is best maintained with continued support following program end. To this end, the KHC encourages participants to enrol in a free 6-month telephone coaching service and facilitates enrolment through an opt-in option at contest registration for participant contact details to be shared with the service. Evaluating participant uptake and outcomes through the coaching service would provide a more complete picture of long-term weight loss outcomes of KHC participants.

### Strengths and limitations

This is the first study to explore weight maintenance among repeat participants of an Aboriginal community-based team weight loss competition. Strengths of this study include the inclusion of 12 contests over 7 years, using a minimum gap of 3 months between participation occasions to allow sufficient time for weight regain, the large sample size, and the inclusion of nutrition and PA behaviours.

Limitations include the ability to measure weight maintenance only among those who repeated the program. The KHC has been shown to be effective for short-term weight loss and healthy lifestyle adaptation [15], and it may be that non-repeaters are able to maintain their weight loss and health behaviours. Among repeaters, we cannot state to what extent the subsequent participation might have mitigated an otherwise larger increase in weight.

## Conclusion

The KHC has been successful in attracting increasingly large numbers of participants, starting with 324 in its first year and increasing to 4433 individuals having participated over the first 7 years of operation. Many participants return to the competition repeatedly, especially women, who had a 34% higher likelihood of repeat than men. Both program weight loss and increases in number of sessions of walking and moderate PA were associated with small (3–7%) but significant increases in likelihood of repeat participation. However, the association between contest weight reduction and subsequent regain (0.05% regain per month for every 1% body weight reduced) might signal a need for the program to incorporate weight loss maintenance strategies to better conserve the benefits accrued during competition. Tracking the longer-term outcomes of those not returning to the competition would assist in understanding the full impact of the KHC. Further, qualitative research with those who do and do not repeat would yield insights into people’s patterns of engagement with the program.

## Supplementary information

**Additional file 1.** Weight_change_among_repeat_Additional_file_1.docx. The additional file contains contest and participation data in tabular form: contest dates (including number of days between each contest), most frequent participation patterns, non-repeater participant characteristics, and further rate ratios for repeat participation.

## Data Availability

The datasets generated and/or analysed during the current study are not publicly available due to the conditions of ethics approval. The contest data that support the findings of this study are not publicly available and restrictions apply to their availability; data may be made available from NSW Ministry of Health, or from the authors upon reasonable request and with permission of NSW Ministry of Health.

## Abbreviations

PA: Physical activity
KHC: NSW Aboriginal Knockout Health Challenge
NSW: New South Wales
kg: Kilogram
sd: Standard deviation
CI: Confidence interval
RR: Rate ratio
