# Supplementary material for: Weight change among repeat participants of an Aboriginal community-based weight loss program
Source: BMC Public Health. 2020 Jun 26;20:1003. doi: 10.1186/s12889-020-09086-6 (PMC7318421; doi:10.1186/s12889-020-09086-6)
Supplement: Supplementary file 1 — Additional file 1. Weight_change_among_repeat_Additional_file_1.docx. The additional file contains contest and participation data in tabular form: contest dates (including number of days between each contest), most frequent participation patterns, non-repeater participant characteristics, and further rate ratios for repeat participation. [file 12889_2020_9086_MOESM1_ESM.docx]

# Additional file for “Weight change among repeat participants of an Aboriginal community-based weight loss program” Bohn-Goldbaum et al

This supplement contains further contest and participation data in tabular form: number of days between each contest, most frequent participation patterns, participant characteristics, and further rate ratios for repeat participation

## Supplemental Table 1. Contest dates

| **Contest** | **Start date** | **End date** | **length (days)** | **days since prior contest** |
| --- | --- | --- | --- | --- |
| 1 | 14/05/2012 | 31/08/2012 | 109 | -- |
| 2 | 4/03/2013 | 28/06/2013 | 116 | 185 |
| 3 | 28/02/2014 | 9/05/2014 | 70 | 245 |
| 4 | 27/06/2014 | 5/09/2014 | 70 | 49 |
| 5 | 2/03/2015 | 22/05/2015 | 81 | 178 |
| 6 | 15/06/2015 | 4/09/2015 | 81 | 24 |
| 7 | 4/04/2016 | 17/06/2016 | 74 | 213 |
| 8 | 4/07/2016 | 16/09/2016 | 74 | 17 |
| 9 | 3/04/2017 | 9/06/2017 | 67 | 199 |
| 10 | 3/07/2017 | 8/09/2017 | 67 | 24 |
| 11 | 26/03/2018 | 1/06/2018 | 67 | 199 |
| 12 | 3/09/2018 | 9/11/2018 | 67 | 94 |
| average |  |  | 75.8 | 129.7 |

## Supplemental Table 2. Participation Patterns

| **Pattern** | **N** | **% participants** |
| --- | --- | --- |
| 000010000000 | 384 | 8.66 |
| 001000000000 | 382 | 8.62 |
| 000000000010 | 305 | 6.88 |
| 010000000000 | 296 | 6.68 |
| 000000001000 | 287 | 6.47 |
| 000001000000 | 251 | 5.66 |
| 000000100000 | 230 | 5.19 |
| 000000000001 | 221 | 4.99 |
| 000100000000 | 177 | 3.99 |
| 100000000000 | 156 | 3.52 |
| 000000000100 | 137 | 3.09 |
| **000000000011** | 85 | 1.92 |
| **(continued)** | **N** | **% participants** |
| **000000001100** | 82 | 1.85 |
| **001100000000** | 81 | 1.83 |
| **000000110000** | 76 | 1.71 |
| 000000010000 | 75 | 1.69 |
| **000011000000** | 43 | 0.97 |
| **110000000000** | 41 | 0.92 |
| **011000000000** | 36 | 0.81 |
| **000000101000** | 27 | 0.61 |
| **000000000111** | 24 | 0.54 |
| **000000001010** | 24 | 0.54 |
| **000000001111** | 22 | 0.50 |
| **000010001000** | 21 | 0.47 |
| **001010000000** | 21 | 0.47 |

The 25 most frequent participation patterns in contests 1-12 are shown, where each digit represents a contest: 1=participated and 0 = no participation. Repeat patterns are shown in bold. The number of individuals in the pattern and the % of total participants are shown in the 2^nd^ and 3^rd^ columns, respectively.

## Supplemental Table 3. Participant characteristics (adding non-repeaters to manuscript’s Table 1)

| Baseline characteristic | | All participants | | | | Repeaters only | | | | Non-repeaters | | | |
| --- | --- | --- | --- | --- | --- | --- | --- | --- | --- | --- | --- | --- | --- |
|  | | mean ± sd | | n (%) | | mean ± sd | | n (%) | | mean ± sd | | n (%) | |
| Total | |  | | 4433 (100%) | |  | | 1,532 (100%) | |  | | 2,901 (100%) | |
| Males | |  | | 1174 (26%) | |  | | 352 (23%) | |  | | 822 (28%) | |
| Age in years | | 38.3 ± 12.95 | | 4387 | | 38.2 ± 12.15 | | 1529 | | 38.4 ±13.36 | | 2858 | |
| Weight in kg | | 96.6 ± 23.52 | | 4403 | | 97.1 ± 22.62 | | 1524 | | 96.4 ±23.99 | | 2879 | |
| Number of fruit serves | | 1.4 ± 1.17 | | 3819 | | 1.45 ± 1.16 | | 1264 | | 1.4 ±1.17 | | 2555 | |
| Number of vegetable serves | | 2.1 ± 1.52 | | 3932 | | 2.1 ± 1.48 | | 1304 | | 2.1 ±1.54 | | 2628 | |
| Number of sessions vigorous PA | | 1.8 ± 2.40 | | 3515 | | 1.9 ± 2.68 | | 1148 | | 1.7 ±2.26 | | 2367 | |
| Number of sessions walking | | 2.3 ± 2.71 | | 3524 | | 2.2 ± 2.41 | | 1151 | | 2.3 ±2.84 | | 2373 | |
| Number of session moderate PA | | 1.6 ± 2.19 | | 3494 | | 1.6 ± 2.04 | | 1140 | | 1.6 ±2.27 | | 2354 | |
|  |  | |  | |  | |  | |  | |  | |  |

Baseline was defined as an individual’s first participation occasion. Analysis included data for 4433 participants, of whom 1532 participated more than once, using a complete case approach.

## Supplemental Table 4. **Rate ratio for repeat participation in KHC for meeting dietary and PA recommendations at first participation start (bivariate analyses)**

| Baseline characteristic,  Meeting recommendation for… | Rate ratio (RR)* | 95%CI | p value |
| --- | --- | --- | --- |
| Fruit | 1.07 | (0.95, 1.20) | 0.27 |
| Vegetable | .99 | (0.80, 1.23) | 0.96 |
| PA | 1.06 | (0.94, 1.20) | 0.32 |

* compared to not meeting recommendations. As a complete case approach was used, the n for each analysis varies: fruit=3,494, vegetable=3,607, PA=3194.
